# Biosensor-Assisted Adaptive Laboratory Evolution for Violacein Production

**DOI:** 10.3390/ijms22126594

**Published:** 2021-06-19

**Authors:** Da-ae Gwon, Joo Yeon Seok, Gyoo Yeol Jung, Jeong Wook Lee

**Affiliations:** 1 Department of Chemical Engineering, Pohang University of Science and Technology, 77 Cheongam-ro, Nam-gu, Pohang, Gyeongbuk 37673, Korea; kda1230@postech.ac.kr (D.G.); gyjung@postech.ac.kr (G.Y.J.); 2 School of Interdisciplinary Bioscience and Bioengineering, Pohang University of Science, 77 Cheongam-ro, Nam-gu, Pohang, Gyeongbuk 37673, Korea; jyseok@postech.ac.kr

**Keywords:** violacein, tryptophan, adaptive laboratory evolution, biosensor

## Abstract

Violacein is a naturally occurring purple pigment, widely used in cosmetics and has potent antibacterial and antiviral properties. Violacein can be produced from tryptophan, consequently sufficient tryptophan biosynthesis is the key to violacein production. However, the complicated biosynthetic pathways and regulatory mechanisms often make the tryptophan overproduction challenging in *Escherichia coli*. In this study, we used the adaptive laboratory evolution (ALE) strategy to improve violacein production using galactose as a carbon source. During the ALE, a tryptophan-responsive biosensor was employed to provide selection pressure to enrich tryptophan-producing cells. From the biosensor-assisted ALE, we obtained an evolved population of cells capable of effectively catabolizing galactose to tryptophan and subsequently used the population to obtain the best violacein producer. In addition, whole-genome sequencing of the evolved strain identified point mutations beneficial to the overproduction. Overall, we demonstrated that the biosensor-assisted ALE strategy could be used to rapidly and selectively evolve the producers to yield high violacein production.

## 1. Introduction

Violacein is one of the tryptophan derivatives and has attracted attention in the pharmaceutical industry due to its antibacterial, antiviral, antifungal, and antitumor activity [1,2]. In addition, it has potential uses as a purple pigment in the cosmetic and textile industries [3]. In order to obtain violacein, natural violacein-producing microorganisms such as *Chromobacterium violaceum* and *Janthinobacterium lividum* have been used. However, these natural producers are pathogenic, and genetic engineering tools are insufficient, so development of such producers is limited [4,5,6]. Alternatively, conventional model organisms such as *Escherichia coli* have been used to construct heterologous violacein biosynthetic pathways [7,8]. In particular, studies using carbon sources such as glucose or glycerol for violacein production have been mainly reported elsewhere, but no studies focused on galactose as a sole carbon source. As one of the alternative carbohydrates, galactose is commonly found in non-edible biomass macroalgae and dairy waste [9,10,11] and is often considered a promising carbon source for biochemical production.

**Figure 1:**
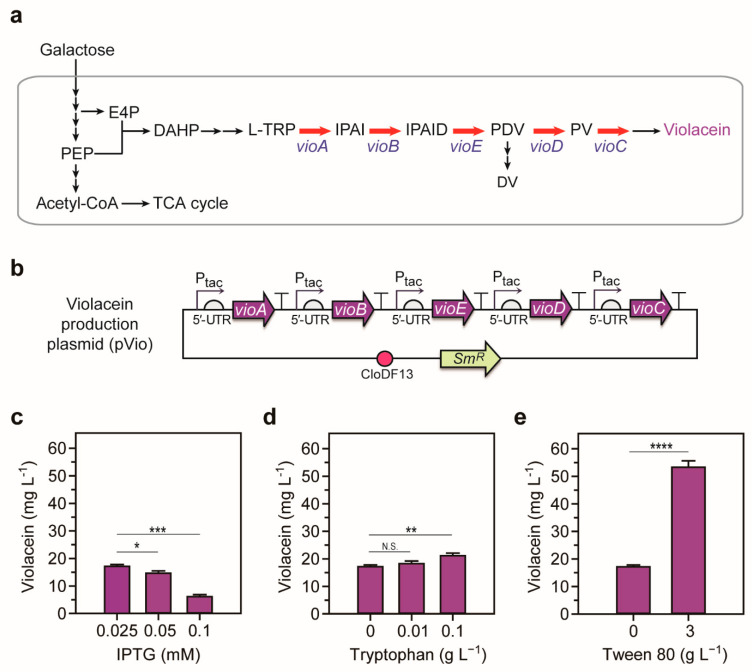
Biosynthesis of violacein in *E. coli*. (**a**) Synthetic pathway for violacein production from galactose in recombinant *E. coli* used in this study. E4P, erythrose 4-phosphate; PEP, phosphoenolpyruvate; DAHP, 3-deoxy-d-arabinoheptulosonate 7-phosphate; L-TRP, L-tryptophan; IPAI, indole-3-pyruvic acid imine; IPAID, indole-3-pyruvic acid imine dimer; PDV, prodeoxyviolacein; PV, proviolacein; DV, deoxyviolacein; 5′-UTR, 5′-untranslated region. (**b**) A schematic diagram of violacein production plasmid (pVio). The genes of *vioABCDE* derived from *C. violaceum* were arranged monocistronically with tac promoter in pCDFDuet-1 vector. The 5′-UTRs were designed using UTR Designer to achieve maximum translation efficiency [30]. (**c**–**e**) Fermentation condition tests in small-scale liquid culture. IPTG concentration (**c**), tryptophan concentration (**d**), and surfactant (**e**). All tests were performed with three biological replicates (two-tailed Student’s *t*-test; N.S. (Not significant) *p* > 0.05; * *p* < 0.05; ** *p* < 0.01; *** *p* < 0.001; **** *p* < 0.0001; Error bars represent ± standard deviation).

The violacein production pathway from galactose can be divided into a tryptophan production pathway from galactose and a violacein production pathway from tryptophan (Figure 1a). For efficient violacein production, the flux of each pathway should be sufficiently obtained. However, the tryptophan biosynthesis/degradation pathways are intertwined with essential metabolic pathways for cell growth and are controlled by various regulatory mechanisms. These features make tryptophan production relatively challenging. In this case, evolutionary approaches with relatively low knowledge dependence can be alternatives [12,13]. Among those, adaptive laboratory evolution (ALE) allows genome-wide optimization of strains by detouring sophisticated genetic manipulation [14,15,16,17,18,19,20]. Moreover, evolution can be facilitated by employing a specific biosensor for the target product [21,22,23,24]. Genetically encoded biosensors can recognize ligands such as metabolites [12,25,26]. These biosensors, often connected to antibiotic resistance genes, are used to enrich cells that produce target metabolites under selection pressure, i.e., antibiotics [27,28,29]. As a result, cells can evolve spontaneously toward obtaining optimal genotypes for target production.

In this study, we developed *E. coli* that can effectively produce violacein by metabolizing galactose. Synthetic expression cassettes were introduced to maximize the synthetic violacein pathway, and culture conditions were evaluated. Subsequently, strain improvement of an evolutionary approach was performed to acquire a sufficient precursor pool, and a sensor consisting of *tnaC* recognizing tryptophan and tetracycline resistance marker was developed. Connecting this sensor to the adaptive laboratory evolution strategy allowed selection of strains that produced tryptophan well. Consequently, we obtained a strain that could effectively metabolize galactose and produce tryptophan, resulting in increased violacein production. Whole-genome sequencing of the evolved strain identified beneficial point mutations for the overproduction.

## 2. Results

### 2.1. Optimization of the Violacein Production System

Since violacein can be synthesized from tryptophan through five enzymatic reactions catalyzed by VioA, VioB, VioC, VioD, and VioE [31], sufficient tryptophan generation would be the key for the violacein overproduction. To do so, we sought to use a previously reported microbial tryptophan producer [32,33,34,35]. Among them, we chose ATCC31743, which has a recombinant plasmid that overexpresses tryptophan biosynthesis genes but lacks genes involved in tryptophan degradation (*tnaA*) and feedback inhibition (*trpR*). This strain produced 3.1 g L^−1^ tryptophan for 48 h [36]. We replaced the antibiotic resistant marker of the tryptophan overproducing plasmid to *kanR* and named the strain EPW.

Using the EPW as a base strain, we constructed a violacein biosynthetic pathway derived from a natural violacein producer, *C. violaceum* (Figure 1a) [1,37]. The genes were expressed monocistronically in a medium-copy number plasmid to minimize metabolic burden (Figure 1b). Each gene was overexpressed using a strong inducible promoter P_tac_, and the 5′-UTR was redesigned using UTR Designer to achieve maximum translation efficiency [30].

Since galactose has been rarely used as a carbon source in microbial violacein production, we explored violacein production conditions by adjusting several parameters, including inducer concentrations, supplemented tryptophan concentration, and surfactant concentration. First of all, we examined violacein production by varying the concentration of inducer, Isopropyl β-d-1-thiogalactopyranoside (IPTG) (Figure 1c). Among the 0.025, 0.05, and 0.1 mM of IPTG tested, the violacein production increased with decreasing IPTG concentration, and 0.025 mM of IPTG showed the highest violacein concentration. This suggests that adequate expression of *vioABCDE* genes is necessary to yield high violacein production as reported elsewhere [8]. We then tested the effect of additional tryptophan supplementation for violacein production. Compared to no additional supplementation, violacein production increased by 1.23-fold when 0.1 g L^−1^ of tryptophan was added (Figure 1d). The amount of tryptophan, the key precursor of the violacein production, affected violacein production positively, indicating that additional generation of tryptophan would be helpful to enhance violacein production. Lastly, we tested the effect of surfactant on violacein production. In previous studies, it has been reported that the addition of surfactant to the culture medium can improve tryptophan production by changing the efflux of aromatic amino acids through affecting membrane fluidity and permeability [38]. Since tryptophan productivity was the highest when Tween 80 was supplemented among several surfactants (Tween 80, PL61, PEG-10000, Triton X-114, Polyvinyl alcohol, and Span80) [38], we introduced Tween 80 to the culture medium. When 3 g L^−1^ of Tween 80 was added, the violacein production increased by 3.11-fold compared to 0 g L^−1^ of Tween 80 (Figure 1e), indicating that the use of surfactant is beneficial for the violacein production.

### 2.2. Development of Tryptophan-Responsive Sensors

**Figure 2:**
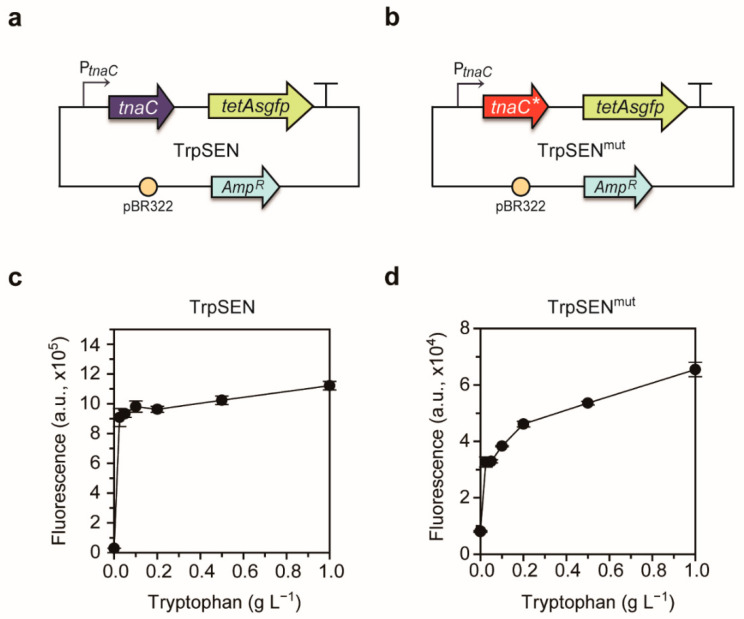
Construction of tryptophan biosensor. (**a**) A schematic diagram of the tryptophan biosensor named TrpSEN. (**b**) A schematic diagram of the engineered tryptophan biosensor named TrpSEN^mut^, in which the 21st codon (aspartic acid, D) of *tnaC*, was replaced by threonine, T. (**c**) Dose-response curve of the TrpSEN containing the wild-type *tnaC*. The GFP fluorescence was measured using a flow cytometry. The *y*-axis represents mean value of sGFP, and the *x*-axis represents tryptophan concentrations (0, 0.025, 0.05, 0.1, 0.2, 0.5, and 1 g L^−1^) added to the culture medium. (**d**) Dose-response curve of the TrpSEN^mut^ containing the mutant *tnaC*. Error bars represent ± standard deviation in duplicate experiments.

As shown in Figure 1d, tryptophan supplementation improved violacein production. We hypothesized that engineering the cells to effectively metabolize galactose would yield higher violacein production. Since galactose is a non-preferred sugar and regulations of tryptophan biosynthesis/degradation pathways are complicated, an evolutionary engineering strategy was introduced rather than rational engineering approaches. In particular, we implemented an ALE strategy using a biosensor, which can accelerate the acquisition of genome-wide spontaneous and positive mutations in the evolution process [39]. First, we developed a biosensor that induces the expression of a fluorescent reporter and tetracycline-resistance gene as a selection marker upon recognition of tryptophan. The biosensor is composed of *E. coli tnaC* gene, encoding the TnaC leader peptide, which attenuates the *tnaAB* expression in the absence of tryptophan [40]. Thus, we fused the *tnaC* gene with *tetA*-*sgfp* gene using primers listed in Table 1 and named TrpSEN (Figure 2a). In the absence of tryptophan, the ribosome stalls at the final codon, P24 in TnaC, and rho factor approaches the ribosome, terminating the transcription before reaching the downstream gene, *tetA*-*sgfp* [41]. In the presence of tryptophan, however, tryptophan binds to the exit tunnel in the ribosome, resulting in the interaction between TnaC and the ribosome. In this case, the rho termination factor cannot approach the ribosome, and consequently, the downstream fused reporter gene can be fully transcribed. Accordingly, the expression of the selection marker *tetA* encoding a tetracycline/H+ antiporter was induced by tryptophan, allowing only tryptophan-producing cells to survive in the tetracycline-containing culture medium.

We examined the performance of the sensor plasmid, TrpSEN, at the fluorescence level using a flow cytometer and found that the sensor showed a narrow operational range from 0 to 0.025 g L^−1^ of tryptophan (Figure 2c, Supplementary Figure S1a). The sensor was too sensitive to the changes of tryptophan with the narrow operational range, and thus unsuitable for adaptive evolution. To expand the operational range, the tryptophan-responsive sensor TnaC was modified. Based on the previous study, D21T substitution of TnaC lowers the affinity between tryptophan and broadens the operational range [41], hence we introduced this mutation to obtain TrpSEN^mut^ (Figure 2b, Supplementary Figure S1b). The TrpSEN^mut^, fluorescence gradually increased as the concentration of tryptophan was changed from 0 to 1 g L^−1^, indicating a wider operational range than the TrpSEN (Figure 2d). Therefore, we determined to use TrpSEN^mut^ for the adaptive evolution of EPW.

### 2.3. Tryptophan-Responsive Sensor-Driven ALE for Violacein Overproduction

**Figure 3:**
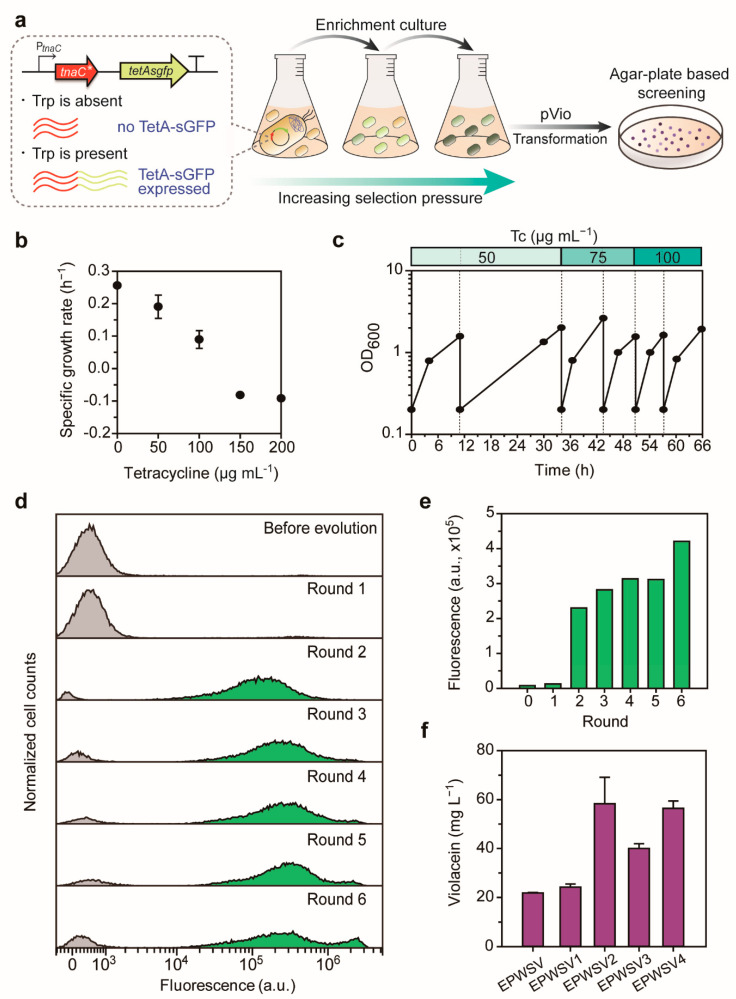
Tryptophan sensor-driven ALE. (**a**) A schematic diagram of the ALE performed in this study. (**b**) Specific growth rates of EPWS, i.e., EPW containing TrpSEN^mut^, were measured with various concentrations of tetracycline to determine the initial selection pressure. The initial selection pressure was set to 50 µg mL^−1^ of tetracycline. (**c**) Growth profile of EPWS during the adaptive evolution. EPWS was cultured in M9-YE containing galactose by increasing the concentration of tetracycline. Tc, tetracycline. (**d**) Histogram of FITC-channel (excitation filter, 488 nm; emission filter, 525 nm). (**e**) Mean value of sGFP intensity of each round. (**f**) Violacein production of candidate ALE strains in small-scale liquid culture.

During the ALE, we sought to enrich tryptophan-producing cells using the TrpSEN^mut^. With the TrpSEN^mut^, the *tetA* expression is induced by tryptophan, and only cells that produce tryptophan efficiently from galactose survive in the presence of the selective pressure, tetracycline (Figure 3a). Consequently, fractions of high tryptophan-producing cells, which obtain spontaneous and positive mutations for tryptophan overproduction, will be gradually dominant in the culture.

To set up the ALE, we first investigated the tetracycline concentration that hindered the growth of parental strain EPWS, that is EPW containing TrpSEN^mut^ (Figure 3b). The initial selection pressure for the ALE was 50 µg mL^−1^ of tetracycline, at which point the EPWS’s growth was substantially inhibited. In order to give the cells time to adapt to the antibiotic concentration, culture was transferred to the same concentration of antibiotics at least twice. The selection pressure was increased in a stepwise manner to 100 µg mL^−1^, and the EPWS was adaptively evolved during serial passages (Figure 3c). To ensure whether the evolution proceeded well, we periodically collected cells at the end of each round and analyzed the population’s fluorescence using flow cytometry. Notably, the proportion of cells with higher fluorescence increased, and the mean of total fluorescence increased as the enrichment rounds were repeated, indicating cells with tryptophan-producing properties had been enriched during the evolutionary process (Figure 3d,e). After the final round, we collected cells and introduced pVio, the violacein production plasmid, into the cells. Since higher violacein-producing cells show a darker purple color, the darker colonies were selected from all transformants (10^3^–10^4^ colonies, Supplementary Figure S2) and tested in small-scale liquid culture. All candidates showed higher violacein production than the parent, and particularly a strain named EPWSV2 showed 2.7-fold higher concentration (Figure 3f). These results indicate that ALE with the tryptophan-responsive sensor plasmid, TrpSEN^mut^, was successfully implemented.

### 2.4. Examination of the Evolved Strain

**Figure 4:**
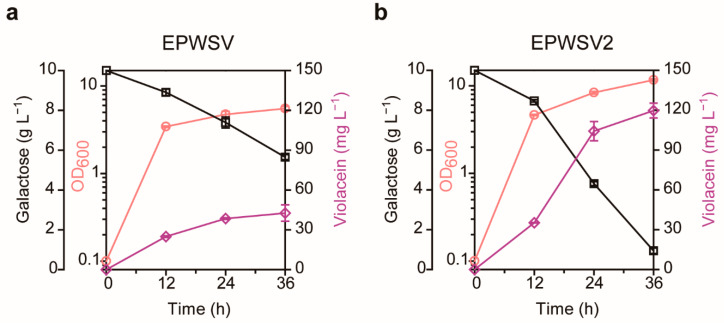
Fermentation profiles of the parental and evolved strains. Physiological comparison between the (**a**) parental strain (EPWSV) and (**b**) evolved strain (EPWSV2) in M9-YE with galactose during 36-h culture. The left *y*-axis and y-offset represent the OD_600_ and the galactose concentration (g L^−1^), respectively. The right *y*-axis represents violacein concentration (mg L^−1^). The *x*-axis represents culture duration (h). The error bars indicate standard deviations of measurements from three biologically independent cultures. Circle, OD_600_; rectangle, galactose; diamond, violacein.

To further analyze the evolved strain, we compared the physiology of the strains in flask culture (Figure 4). Notably, we found that the consumed galactose was more than doubled in the evolved strain named EPWSV2 compared to the parental strain named EPWSV, resulting in a doubled increase in the final biomass. The enhanced cell growth led to an improvement in tryptophan production, which in turn could have contributed to the elevated violacein productivity. In addition, the specific violacein productivity of the EPWSV2 strain showed a 1.33-fold increase compared to that of the EPWSV strain. Moreover, the evolved strain exhibited a 2.81-fold increase in the violacein concentration compared to the parent, yielding 119.73 mg L^−1^ from galactose as a carbon source. Taken together, using the TrpSEN^mut^ containing TnaC^D21T^ as a tryptophan-responsive biosensor, we were able to obtain a strain producing tryptophan by metabolizing galactose, and thereby the productivity of violacein could be effectively improved. In order to identify accumulated mutations in the EPWSV2, whole-genome sequencing was carried out. As a result, 12 mutations were identified, and they were single nucleotide polymorphisms (SNPs). Most of them were revealed as upstream and downstream gene variants, and only five of them were missense variants, which resulted in an amino acid substitution at that position (Table 2). Genes encoding GalR, PtrA, Rnr, RpoC, and TcyP contained a missense mutation. Among the missense mutations, the amino acid change on the GalR occurred within the DNA binding domain, suggesting that the regulation on GalR-regulated genes might be relieved due to the mutation. Consequently, this alleviation of GalR regulation may affect galactose utilization, leading to the overproduction of violacein.

## 3. Discussion

In this study, we reported engineered *E. coli* that can effectively produce violacein by metabolizing galactose. To do so, we first constructed the violacein production pathway in a tryptophan-producing strain with overexpressing heterologous violacein biosynthesis genes. Using the strain, several parameters that could affect violacein production were studied. Among them, we found that additional tryptophan supplementation led to higher violacein production. This result suggested that enhanced tryptophan biosynthesis would be beneficial for the production of violacein.

Since the tryptophan biosynthesis regulatory network is complicated, we took the ALE approach to enhance the tryptophan production. To assist the ALE, we developed a tryptophan-responsive genetic device using *tnaC* that induces reporter expression upon recognition of tryptophan in the cell. The tryptophan-responsive sensor with wild-type *tnaC* showed a narrow operational range, so the *tnaC* was modified to broaden the operational range. It has been reported that the sensitivity of *tnaC* to tryptophan decreases when the 21st amino acid (D, Asp) of *tnaC* is changed to T (Thr) [41]. Based on this, we introduced D21T mutation to the *tnaC* sensor to lower its affinity with tryptophan, and this modification provided a wider operational range of the sensor. Using the optimized tryptophan sensor, we adaptively evolved the parental strain, EPWS, and we then introduced pVio plasmid containing the heterologous violacein pathway to the evolved population to obtain a strain capable of effectively metabolizing galactose and consequently overproducing violacein. Notably, we could instantly screen violacein-overproducing strains on a solid medium with the naked eye due to the distinct color violacein.

The flask cultivation of the evolved strain showed 2.7-fold higher concentration compared to the parent strain, indicating the biosensor-assisted ALE strategy was successful to obtain such an improved strain within a relatively short period. Whole-genome sequencing results identified the evolved strain accumulated a set of mutations that might be related to the production directly and indirectly. Among them, a notable missense mutation was identified on the coding sequence of *galR*, which encodes a DNA-binding transcription factor that inhibits the transcription of operons involved in D-galactose transport and catabolism. In particular, the amino acid change occurred within the DNA binding domain of GalR, suggesting that the regulation on GalR-regulated genes might be alleviated due to the mutation. Therefore, the missense mutation of GalR may affect galactose catabolism and increase galactose utilization, leading to the increased production of violacein (Figure 4).

In conclusion, we implemented the ALE strategy with an aid of the tryptophan-responsive genetic device. This ALE strategy allowed us to detour heavy genomic engineering that is typically required to resolve complicated metabolic regulation. Without the need of targeting specific genes, we could rapidly and selectively evolve the producer in the direction that we aimed. In addition, the resulting strain showed high galactose utilization capability, which can be used to produce other biochemicals, including tryptophan derivatives, from galactose.

## 4. Materials and Methods

### 4.1. Bacterial Strains, Plasmids, and Reagents

The bacterial strains and plasmids used in this study are listed in Table 3. Mach-T1^R^ was used as a host for all plasmid construction. The EPW used for tryptophan production is a strain in which the selectable marker of ATCC31743 has been replaced with a kanamycin resistance gene. Plasmid DNA and genomic DNA were isolated using Exprep™ Plasmid SV kit (GeneAll Biotechnology, Seoul, Korea) and Exgene^TM^ Cell SV kit (GeneAll Biotechnology), respectively. DNA fragments were purified using the Expin™ Gel SV kit (GeneAll Biotechnology). Q5 polymerase and NEBuilder^®^ HiFi DNA Assembly Master Mix were purchased from New England Biolabs (Ipswich, MA, USA). Luria-Bertani (LB) broth and agar used for the cloning process of plasmids, and yeast extract, were obtained from BD Biosciences (Sparks, MD, USA). Other chemicals were attained from Sigma–Aldrich (St. Louis, MO, USA). The oligonucleotides were synthesized by Cosmogenetech (Seoul, Korea) (Table 1).

### 4.2. Construction of Heterologous Violacein Pathway

The violacein production plasmid was constructed using the Gibson assembly method [47]. First, the vector was amplified from pCDFDuet-1 with a set of primers called homology_pCDF_F2/R. *VioA* and *vioB* genes were amplified from pET15b-vioA and pET15b-vioB (GenBank: KX461959 and KX461960) with UTR_vioA_F/term_vioA_R and UTR_vioB_F/term_vioB_R, respectively. To insert a homology sequence at 5′-end, each PCR product was amplified again with homology_vioA_F2/term_vioA_R and homology_vioB_F2/term_vioB_R2, respectively. The fragments of vector, *vioA*, and *vioB* were assembled using the assembly method, and the resultant was named pCPA. To clone the *vioCDE* genes, the vector was first amplified from pCPA with homology_vec_F/R. *VioC*, *vioD* and *vioE* genes were amplified from pET15b-vioC, -vioD, and pET21-vioE (GenBank: KX461961, KX461962 and KX461963) with UTR_vioC_F/term_vioC_R, UTR_vioD_F/term_vioD_R and UTR_vioE_F/term_vioE_R, respectively. The PCR products were also amplified again with homology_vioC_F2/term_vioC_R2, homology_vioD_F2/term_vioD_R, and homology_vioE_F2/term_vioE_R, respectively. The vector containing pCPA and the PCR products of *vioC*, *vioD*, and *vioE* were assembled using the Gibson assembly. All sequences of 5′-UTRs were designed and predicted using the UTR Designer (http://sbi.postech.ac.kr/utr_designer, accessed 1 March 2018) to enhance translation efficiency [30].

### 4.3. Development of Tryptophan-Responsive Sensors

The tryptophan-responsive sensor plasmid was also constructed using the Gibson assembly. The vector was amplified from the pET23b vector with a set of primers named T7_term_F/pET23b_vec_R. A *tnaC* encoding a tryptophan-responsive leader peptide was amplified using a chromosome of *E. coli* MG1655 as a template with a set of primers, tnaC_homo_F/tnaA_homo_w_tetA. The *tetA*-*sgfp* encoding fusion protein was amplified with tetA_F/sfgfp_homo_R. The three PCR amplicons were assembled. To improve sensor performance, a single amino acid mutation was introduced using blunt-end ligation with a primer set of D21T_blunt_F/R.

### 4.4. Validation of Tryptophan-Responsive Sensors

The sensor plasmids for tryptophan detection were transformed into *E. coli* W3110, and the fluorescence intensity was monitored with different tryptophan concentrations. Cells were inoculated into the M9 medium (6.78 g L^−1^ Na_2_HPO_4_, 3 g L^−1^ KH_2_PO_4_, 1 g L^−1^ NH_4_Cl, 0.5 g L^−1^ NaCl, 2 mM MgSO_4_, and 0.1 mM CaCl_2_), supplemented with 50 µg mL^−1^ kanamycin and 100 µg mL^−1^ of carbenicillin for plasmid maintenance. Overnight seeds were inoculated into the fresh medium to make an initial OD_600_ of 0.05. Tryptophan was added to the medium to achieve final concentrations of 0, 0.025, 0.05, 0.1, 0.2, 0.5, and 1 g L^−1^. Fluorescence was analyzed using flow cytometry (CytoFLEX S, Beckman Coulter, Brea, CA, USA) after 6 h of inoculation. Fluorescence of sGFP was measured on a FITC channel, excited with a 488-nm, and detected with a 525/40-nm bandpass filter. At least 20,000 events were recorded per sample.

### 4.5. Adaptive Laboratory Evolution Procedure

To evolve the EPW strain, TrpSEN^mut^ was chosen as a tryptophan sensor, and *tetA*-*sgfp* fusion gene was used as a selection marker. This selection cassette expresses tetracycline resistance gene *tetA*, only in the presence of tryptophan. Using cells at the exponential growth phase (OD_600_ around 0.8), we first tested different tetracycline concentrations from 0 to 200 µg mL^−1^ and found a selection condition under which the cell growth was severely but not completely inhibited. The initial selection condition was 50 µg mL^−1^ of tetracycline. We then raised the tetracycline concentration up to 100 µg mL^−1^ to enrich tryptophan-producing cells but to eliminate other cells. To do so, cells were cultured in 2 mL of M9-YE (M9 medium containing 1g L^−1^ yeast extract) with an initial OD_600_ of 0.05. When OD_600_ reached 0.8, 50 µg mL^−1^ of tetracycline was added to the culture, and cells were cultured until it reached OD_600_ of 2.0. The cells were diluted into a fresh medium, and the enrichment procedure was repeated for six rounds. During the enrichment procedure, tetracycline was added at a concentration of 50, 50, 75, 75, 100, and 100 µg mL^−1^.

### 4.6. Culture Conditions for Violacein Production

All cultivation experiments for violacein production were performed using the modified M9-YE medium containing 10 g L^−1^ galactose with appropriate antibiotics. To produce violacein, a single colony was obtained from a streak plate. The single colony was inoculated into the M9-YE medium. The overnight culture was inoculated into the fresh M9-YE medium containing 0.025 mM of IPTG and with an initial OD_600_ of 0.05. The cells were incubated at 37 °C for 4 h, and the temperature was lowered to 30 °C. When tested in small-scale liquid culture, 2 mL of M9-YE medium without surfactant was used. A 250 mL baffled flask containing 20 mL of M9-YE medium 3 g L^−1^ Tween 80 was used, and the agitation speed for fermentation was 200 rpm.

### 4.7. Analytical Methods

For the measurement of crude violacein, cells were first collected by centrifugation. After removing the supernatant, violacein was extracted by mixing the collected cells with ethanol in an ultrasonic water bath (Cpx5800h-e, Branson Ultrasonics Corporation, Danbury, CT, USA) at 60 °C until the cells were fully bleached. All ethanol extracts were collected, and the absorbance was measured at 570 nm to quantify the violacein using a Hidex Sense 425-301 microplate reader (HIDEX, Turku, Finland). The concentration of violacein was calculated based on a standard curve prepared using the purchased violacein (Sigma-Aldrich). In order to accurately quantify violacein in the flask fermentation, we used analytical high-performance liquid chromatography (HPLC) system (UltiMate^TM^ 3000 analytical HPLC system, Dionex, Sunnyvale, CA, USA) equipped with Acclaim 120 C18 reverse-phase column (Dionex). The mobile phases were acetonitrile with 0.1% formic acid (A) and water containing 0.1% formic acid (B). The following gradient was carried out at a flow rate of 1 mL min^−1^: 0 min, 5% A; 1 min, 5% A; 5 min, 45% A; 7 min, 55% A; 9 min, 95% A; 10 min, 5% A; 12 min, 5% A. The signal was detected using an ultraviolet-visible (UV-Vis) diode array detector. Ultraviolet-visible (UV-Vis) diode array detector was used to detect signals. Galactose was analyzed with an Aminex HPX-87H column (Bio-Rad Laboratories, Richmond, CA, USA) maintained at 65 °C. The mobile phase was 5 mM H_2_SO_4_ at a flow rate of 0.6 mL min^−1^. The signals were monitored using a Shodex RI-101 refractive index detector (Shodex, Klokkerfaldet, Denmark).

### 4.8. Whole-Genome Sequencing

The bacterial chromosomal DNAs from EPWSV and EPWSV2 were extracted from an overnight cultured sample using GeneAll Exgene^TM^ Cell SV kit (GeneAll Biotechnology) according to the manufacturer’s protocol. Sequencing libraries were constructed using the TruSeq DNA Nano DNA High Throughput Library Prep Kit (Illumina, San Diego, CA, USA) according to the manufacturer’s instructions. Samples were sequenced using the Illumina NovaSeq 6000 system (DNA Link, Seoul, Korea). Sequencing reads were then mapped onto the reference genome (GCF_000010245.2) using Burrows-Wheeler Aligner (version 0.7.12.) [48].

## Figures and Tables

**Table 1:** Oligonucleotides used in this study.

Name	Sequence (5′-3′)
Primers used for the construction of violacein production plasmid	
homology_pCDF_F2	acaagcttgcggccgcataatgcttaagtc
homology_pCDF_R	attatacgagccgatgattaattgtcaaggatccccatggtatatctccttattaaagttaaacaaaattatttc
UTR_vioA_F	gtgagcggataacaattgaaacggacataaggaggaaattctatgaaacattcttccgatatctgcattg
term_vioA_R	gctgttgcagcgtatcgccgcgtaacgcaaaaaaccccgcttcggcggggttttttcgcgaattcttgac
UTR_vioB_F	tgtgtggaattgtgagcggataacaattaatcgagttcgaaggaggaaaagtcatgagcattctggatttcccgc
term_vioB_R	gcaaactgagccgcgaggcctaacgcaaaaaaccccgcttcggcggggttttttcgcgcggccgcata
homology_vioA_F2	gggatccttgacaattaatcatcggctcgtataatgtgtggaattgtgagcggataacaattgaaacgga
term_vioA_R	gctgttgcagcgtatcgccgcgtaacgcaaaaaaccccgcttcggcggggttttttcgcgaattcttgac
homology_vioB_F2	ttcggcggggttttttcgcgaattcttgacaattaatcatcggctcgtataatgtgtggaattgtgagcggataaca
term_vioB_R2	gcaaactgagccgcgaggcctaacgcaaaaaaccccgcttcggcggggttttttcgcgagctcggcgcgcctgca
homology_vec_F	cctaggctgctgccaccgctgagcaataactagc
homology_vec_R	cttgcggccgcataatgcttaagtcgaaca
UTR_vioC_F	tgtgtggaattgtgagcggataacaattaaggacaacaaaaggaggagaactaatgaaacgtgcgattatcgttggtg
term_vioC_R	ggtacaagattggtcgcgtgaattaacgcaaaaaaccccgcttcggcggggttttttcgc
UTR_vioD_F	tgtgtggaattgtgagcggataacaattccattggagagaaggagggaaattcatgaagattctggtcattggtgctg
term_vioD_R	tgcgttatgctttgcagcgctaacgcaaaaaaccccgcttcggcggggttttttcgcctcgagttg
UTR_vioE_F	aatgtgtggaattgtgagcggataacaattaaaacgaaaataaggaggaaattctatggagaaccgtgagccacc
term_vioE_R	cggttttcgcggccaagcgctaacgcaaaaaaccccgcttcggcggggttttttcgcggtaccttgacaattaatcat cggctcg
homology_vioC_F2	cgcttcggcggggttttttcgcctcgagttgacaattaatcatcggctcgtataatgtgtggaattgtgagcggataac
term_vioC_R2	cttcggcggggttttttcgccctaggctgctgccaccgctgagcaataactagc
homology_vioD_F2	gcggtaccttgacaattaatcatcggctcgtataatgtgtggaattgtgagcggataac
term_vioD_R	tgcgttatgctttgcagcgctaacgcaaaaaaccccgcttcggcggggttttttcgcctcgagttg
homology_vioE_F2	cttgcggccgcataatgcttaagtcgaacattgacaattaatcatcggctcgtataatgtgtggaattgtgagcggataac
term_vioE_R	cggttttcgcggccaagcgctaacgcaaaaaaccccgcttcggcggggttttttcgcggtaccttgacaattaatcatcg gctcg
Primers used for the construction of tryptophan-responsive sensor	
T7_term_F	ctagcataaccccttggggc
pET23b_vec_R	ccgagatctcgatcccgcgaaat
tnaC_homo_F	caacgctgcccgagatctcgatcccgcgaaatgcatgcccgcgcttacgaagccgcattc
tnaA_homo_w_tetA	ccgaggatgacgatgagcgcattgttagatttcatcggttcagggagatgtttaaagttttccattac
tetA_F	atgaaatctaacaatgcgctcatcgtcatc
sfgfp_homo_R	gcatggacgagctgtacaagtaaaccgctgagcaataactagcataaccccttggggc
D21T_blunt_F	acacaccgcccttgatttgccc
D21T_blunt_R	gacaattttgttgtcaatattgaacc

**Table 2:** Accumulated mutations in EPWSV2.

Gene	Function	Position	Mutation		Amino Acid Change
			EPWSV	EPWSV2	
*galR*	DNA-binding transcriptional dual regulator [42]	2975313	G	C	R20P
*ptrA*	Protease 3 [43]	2956475	A	T	S356T
*rnr*	RNase R [44]	4413454	A	C	E707D
*rpoC*	RNA polymerase subunit β’ [45]	3450328	T	A	Q335L
*tcyP*	Cystine/sulfocystein:cation symporter [46]	1812711	A	C	T22P

**Table 3:** Strains and plasmids used in this study.

Name	Relevant Characteristics ^1^	Source
Strains		
Mach1-T1^R^	F^−^ φ80(*lac*Z)ΔM15 Δ*lac*X74 *hsd*R(r_K_^−^m_K_^+^) Δ*rec*A1398 *end*A1 *ton*A	Invitrogen
*E. coli* W3110	F^−^ λ^−^ rph-1 INV(rrnD, rrnE)	ATCC39936
ATCC31743	*E. coli* W3110*-*Δ*trpABCDE*-Δ*trpR*-Δ*tnaA*/pSC101-*trpABCDE*	ATCC31743
EPW	ATCC31743, Kan^R^	This study
EPWS	EPW/TrpSEN^mut^	This study
EPWSV	EPWS/pVio	This study
EPWSV1	Evolved strain derived from EPWSV	This study
EPWSV2	Evolved strain derived from EPWSV	This study
EPWSV3	Evolved strain derived from EPWSV	This study
EPWSV4	Evolved strain derived from EPWSV	This study
Plasmids		
pCDFDuet-1	Expression vector, Sm^R^, CloDF13 ori	Novagen
pET-23b	Expression vector, Amp^R^, pBR322 ori	Novagen
pCPA	pCDF-P_tac_::synUTR_vioA_-*vioA*::Ter- P_tac_::synUTR_vioB_-*vioB*::Ter	This study
pVio	pCDF-P_tac_::synUTR_vioA_-*vioA*::Ter- P_tac_::synUTR_vioB_-*vioB*::Ter-P_tac_::synUTR_vioE_-*vioE*::T-P_tac_::synUTR_vioD_-*vioD*::Ter-P_tac_::synUTR_vioC_-*vioC*::Ter	This study
TrpSEN	pET-23b-P_tnaC_::*tnaC*-*tetA*-*sfgfp*::Ter	This study
TrpSEN^mut^	pET-23b-P_tnaC_::*tnaC*(D21T)-*tetA*-*sfgfp*::Ter	This study

^1^ Abbreviations: Amp, Ampicillin; Kan, Kanamycin; Sm, Streptomycin; R, resistance; *E. coli*, *Escherichia coli*; Ter, Terminator.

## Data Availability

Not applicable.

## Supplementary Materials

The following are available online at https://www.mdpi.com/article/10.3390/ijms22126594/s1.

## Abbreviations

ALE	Adaptive Laboratory Evolution
*E. coli*	*Escherichia coli*
*C. violaceum*	*Chromobacterium violaceum*
E4P	Erythrose 4-phosphate
PEP	Phosphoenolpyruvate
DAHP	3-deoxy-d-arabinoheptulosonate 7-phosphate
L-TRP	L-tryptophan
IPAI	indole-3-pyruvic acid imine dimer
PDV	prodeoxyviolacein
PV	proviolacein
DV	deoxyviolacein
5′-UTR	5′-untranslated region
SNP	single nucleotide polymorphism
