# Supplementary material for: Biosensor-Assisted Adaptive Laboratory Evolution for Violacein Production
Source: Int J Mol Sci. 2021 Jun 19;22(12):6594. doi: 10.3390/ijms22126594 (PMC8233975; doi:10.3390/ijms22126594)
Supplement: Supplementary file 1 [file ijms-22-06594-s001.zip › ijms-1234719-supplementary.pdf]

Supplementary Information

Biosensor-assisted adaptive laboratory evolution for violacein production

Da-ae Gwon, Joo Yeon Seok, Gyoo Yeol Jung, and Jeong Wook Lee

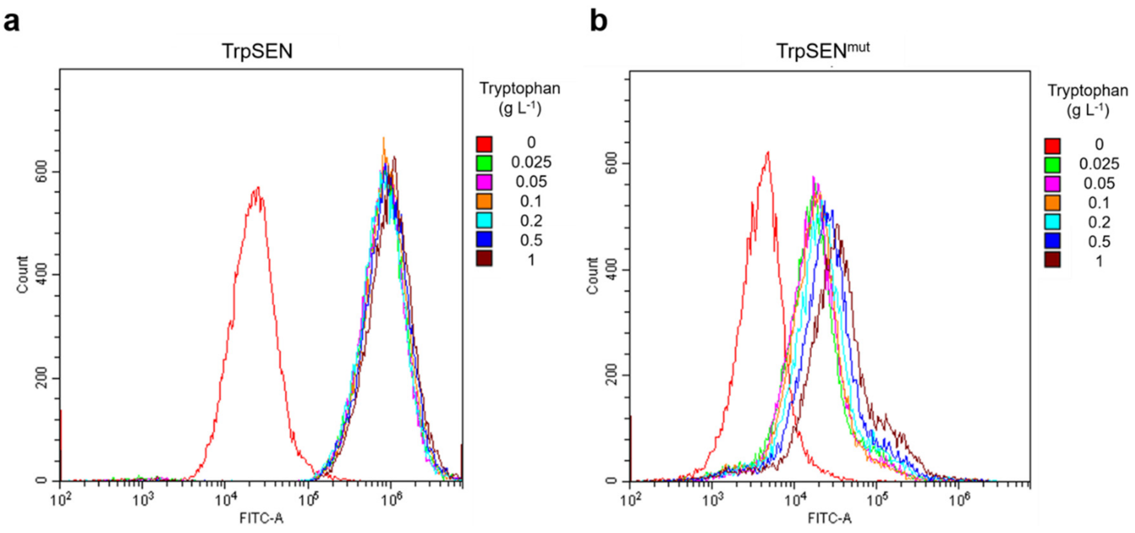

Supplementary Figure S1. FITC Histogram according to tryptophan concentration. (**a**) TrpSEN containing the wild-type *tnaC* (**b**) TrpSEN^{mut} containg mutant *tnaC*

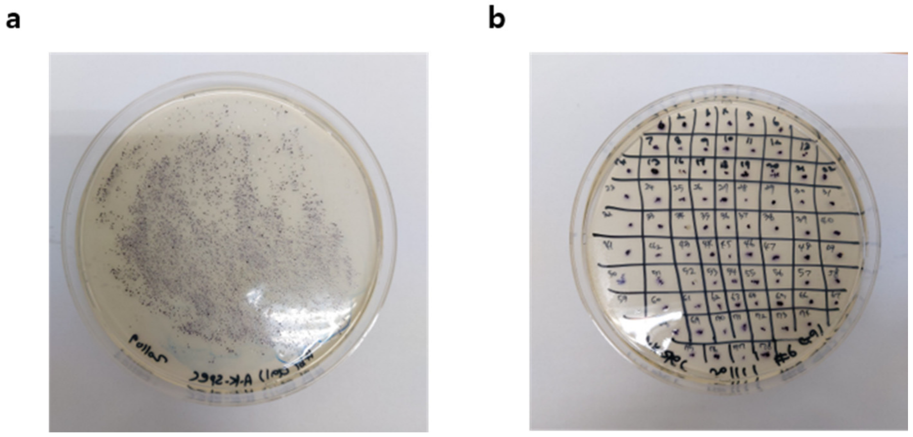

Supplementary Figure S2. Photo of colonies after transformation (**a**) Colonies after transformation of pVio into EPWSV2 (**b**) Among 70 colonies were selected
